# HSTXGB: a hyperparameter self-tuning XGBoost method integrating pre- and post-processing for gene regulatory network inference

**DOI:** 10.3389/fgene.2026.1895515

**Published:** 2026-09-04

**Authors:** Mingqing Huang, Shun Guo, Fengze Jiang, Yingnan Xiong, Xu Chen

**Affiliations:** 1 School of Computer and Software, Shenzhen University of Information Technology, Shenzhen, China; 2 Shenzhen Institute of Advanced Technology, Chinese Academy of Sciences, Shenzhen, China; 3 School of Artificial Intelligence, Shenzhen University of Information Technology, Shenzhen, China

**Keywords:** gene regulatory expression data, gene regulatory network, hyperparameter self-tuning, regulatory ranking inference, regulatory structure refinement, XGBoost

## Abstract

Clarifying gene regulatory networks (GRNs) remains one of the central challenges of systems biology and is crucial for elucidating pathogenesis and curing diseases. Various machine learning techniques have been developed for gene regulatory network inference, but identifying intricate interactions is still a fundamental problem. Here, we propose a network structure refinement scheme, termed HSTXGB (a hyperparameter self-tuning XGBoost method integrating pre- and post-processing), to infer GRNs from time-course expression data by leveraging the nonlinear modeling capability of XGBoost while integrating prior knowledge (e.g., knockout data) and posterior statistics (e.g., regulation probabilities). Specifically, HSTXGB first calculates regulation relationship confidences using a self-tuning XGBoost model, which accounts for temporal dependencies in gene expression. Then, two novel strategies are designed to integrate information from prior data and to incorporate statistical information, which correspond to fluctuations in knockout experiments and to regulatory frequency and intensity, respectively. The confirmatory experiments on the benchmark datasets from the DREAM challenge as well as the *E. coli* datasets (8 networks in total) demonstrated that our HSTXGB scheme achieves significantly better performance compared with eight other state-of-the-art methods.

## 1 Introduction

Gene regulatory networks (GRNs) consist of molecular regulators that interact with each other to determine gene activation and silencing in specific cellular contexts. Elucidating GRNs facilitates the analysis and understanding of biological development, function, and pathology—for instance, by explaining how cells perform diverse functions, how they modulate gene expression in response to environmental changes, and how noncoding genetic variants contribute to disease. Consequently, GRN inference has emerged as a fundamental task in bioinformatics (Yuan and Zhana, 2025; Liu et al., 2023).

The advent of high-throughput experimental techniques such as RNA-Seq (Wang et al., 2009) and DNA microarrays (Bumgarner, 2013) has made it feasible to infer GRNs from the resulting data at the genomic scale. Accordingly, the increasing accessibility of gene expression datasets (Greenfield et al., 2010; Jozefczuk et al., 2010) has spurred the emergence of diverse computational strategies (Marku and Pancaldi, 2023) aimed at reconstructing network architectures. These reverse-engineering approaches are generally organized into four distinct methodological classes: model-based, edge importance-based, network topology-based, and feature selection-based methods (Liu et al., 2023).

Model-based methods infer GRNs using complex computational models, such as Boolean models (Pušnik et al., 2022), Bayesian networks (Liu et al., 2016), and differential equation models (Yang and Chen, 2020). Boolean models represent gene expression levels as discrete dynamic systems with two possible states: on and off. While conceptually simple, they are limited to capturing only basic regulatory interactions and often fail to reflect the complex regulatory mechanisms inherent in GRNs. Bayesian networks are probabilistic graphical models capable of capturing both the stochastic nature of biological systems and the causal relationships in gene regulation. However, their application to large-scale GRN inference is constrained by the substantial increase in computational complexity with network size. Differential equation models describe regulatory interactions between genes through differential equations. They effectively capture the continuity of expression data and accurately model the dynamic processes of gene expression changes, making them well suited for simulating nonlinear dynamic systems. Nevertheless, given the diversity of gene expression data types and the complexity of regulatory mechanisms, it remains challenging to define a stable set of equations with appropriate parameters for GRN inference.

Edge importance-based methods treat each edge as the basic unit of GRN inference. They simplify the inference process by directly computing and ranking the importance scores between gene pairs. Representative measures include Pearson Correlation Coefficient (PCC) (Salleh et al., 2015), Euclidean Distance (ED) (Ghosh and Barman, 2016), Partial Correlation (PC) (Watson-Haigh et al., 2010), and Mutual Information (MI) (Tabbaa and Jayaprakash, 2014). PCC and ED are widely used to evaluate linear relationships between genes, but they fail to distinguish indirect associations from direct ones. PC addresses this limitation by incorporating conditional information, yet it struggles to detect nonlinear relationships (Leng et al., 2020; Werme et al., 2022). In contrast, MI, grounded in information theory, is considered a nonlinear extension of correlation measures (Pušnik et al., 2022). Several improved variants, such as Conditional MI (CMI) (Xu et al., 2022), Conditional Mutual Inclusive Information (CMI2) (Lei et al., 2023), and Part MI (PMI) (Jiang et al., 2022), aim to more accurately quantify regulatory strengths between genes. However, none of these measures can infer the direction of gene regulation. Consequently, some researchers have turned to alternative perspectives for GRN inference, particularly those based on feature selection.

Network topology-based methods integrate network topology algorithms with edge importance measures for GRN inference. Among these, the Path Consistency Algorithm (PCA) (Mohr and Henderson, 1986) is widely adopted due to its particular effectiveness in sparse and scale-free networks. Representative methods based on PCA include PCA-CMI (Zhang et al., 2012) and CMI2NI (Zhang et al., 2015). Nevertheless, these methods still suffer from certain limitations. For instance, PCA initializes GRN inference with an undirected complete graph, meaning that all edges are assumed to be true by default. Consequently, only those redundant edges that can be identified by the importance measure are removed, while other redundant edges tend to persist in the network. Moreover, these methods typically emphasize local neighborhood information rather than the global network topology, which is insufficient for fully exploiting the topological properties of GRNs. To address these issues, RWRNET (Liu et al., 2020) employs a random walk with restart algorithm to capture global network topology, and NSCGRN (Liu et al., 2022) adopts a network structure control method that defines specific forms and cooperative modes for both global and local topologies. Although these two methods effectively reduce redundant edges, they cannot produce a ranked list of gene regulatory interactions due to their reliance on network topologies. Subsequently, NSRGRN (Liu et al., 2023) constructs a preliminary ranking of gene regulatory relationships and develops a novel network structure refinement algorithm to optimize the network architecture. However, its performance on benchmark datasets still requires further improvement.

Feature selection-based methods decompose the network inference problem involving numerous genes into smaller sub-problems. In this framework, each sub-problem designates a single gene as the target and focuses exclusively on regulatory interactions directed from candidate regulators toward that target. Among such methods, GENIE3 (Huynh-Thu et al., 2010), which employs ensembles of random forests, is considered state-of-the-art on several benchmarks. Its dynamic extension, dynGENIE3 (Huynh-Thu and Geurts, 2018), is capable of handling both time-series and steady-state expression data. However, a single feature selection algorithm often struggles to accurately characterize the importance of all edges, leading to the presence of highly ranked but redundant regulatory relationships. To address this limitation, methods such as NIMEFI (Ruyssinck et al., 2014) and MMFGRN (He et al., 2021) attempt to leverage the combined strengths of multiple algorithms (Che et al., 2020). NIMEFI provides a general framework that converts a feature selection algorithm into an ensemble version using a subsampling strategy. Its core idea is to use rank-wise averaged predictions from multiple ensemble runs as the final ranking of regulatory links. Similarly, MMFGRN simultaneously integrates different data types and models by fusing three distinct inference approaches: a single time-series data model, a single steady-state data model, and a joint model that incorporates both time-series and steady-state data. The first two models are built using the LightGBM algorithm (Ke et al., 2017), while the third employs XGBoost (Chen and Guestrin, 2016). Although NIMEFI and MMFGRN have achieved promising results, they typically involve a large number of tunable parameters, which ultimately increases the uncertainty of the predictions.

In this study, we propose a feature selection-based method, termed HSTXGB—a hyperparameter self-tuning XGBoost approach that integrates both pre-processing and post-processing steps—for the inference of gene regulatory networks. This method achieves near-optimal performance by automatically tuning the hyperparameters of XGBoost to handle time-series genetic data, while additionally incorporating pre-processing of knockout data and post-processing of statistical information. The main contributions and characteristics of our framework are summarized as follows:

1. By utilizing XGBoost, our model effectively captures nonlinear and complex regulatory relationships within gene expression profiles, while simultaneously providing reliable feature importance rankings.
2. We adopt a hyperparameter self-tuning strategy, which adaptively configures the model’s hyperparameters for each target gene individually, thereby ensuring optimal predictive performance.
3. We further enhance the model by integrating pre-processed knockout data and post-processed statistical information, which together help to better harness the regulatory confidence among genes and improve overall inference accuracy.

## 2 Materials and methods

The benchmark datasets DREAM challenge (Greenfield et al., 2010) and *E. coli* (Jozefczuk et al., 2010), widely recognized as public standard gene expression datasets, were adopted in our study for method validation and comparison.

XGBoost (Chen and Guestrin, 2016), known for its excellent performance in numerical prediction, was integrated with pre- and post-processing and equipped with hyperparameter self-tuning to identify gene regulatory relationships in this study.

### 2.1 Benchmark datasets

#### 2.1.1 DREAM4 *in silico* size-100 challenge networks

The DREAM4 *in silico* 100-gene challenge dataset comprises five distinct networks, each consisting of 100 genes with predefined ground-truth regulatory interactions. For each network, both time-series expression profiles and knockout (KO) data are made available to support comprehensive evaluation. The time-series data consists of 10 independent samples, each spanning 21 consecutive time points, thereby enabling the investigation of dynamic regulatory processes and temporal dependencies in gene expression. The knockout data, in contrast, comprises expression measurements obtained by individually knocking out each of the 100 genes, which provides a systematic view of perturbation effects and facilitates the inference of causal regulatory relationships. Together, these two complementary data types offer a robust basis for benchmarking GRN inference algorithms. Table 1 provides a summary of the key statistical characteristics of the dataset.

**TABLE 1:** The details of DREAM4 *in silico* size-100 challenge networks.

Network name	# target genes	# candidate regulators	# samples	# time points	# true edges
Network 1	100	100	10	21	176
Network 2	100	100	10	21	249
Network 3	100	100	10	21	195
Network 4	100	100	10	21	211
Network 5	100	100	10	21	193

**FIGURE 1:**
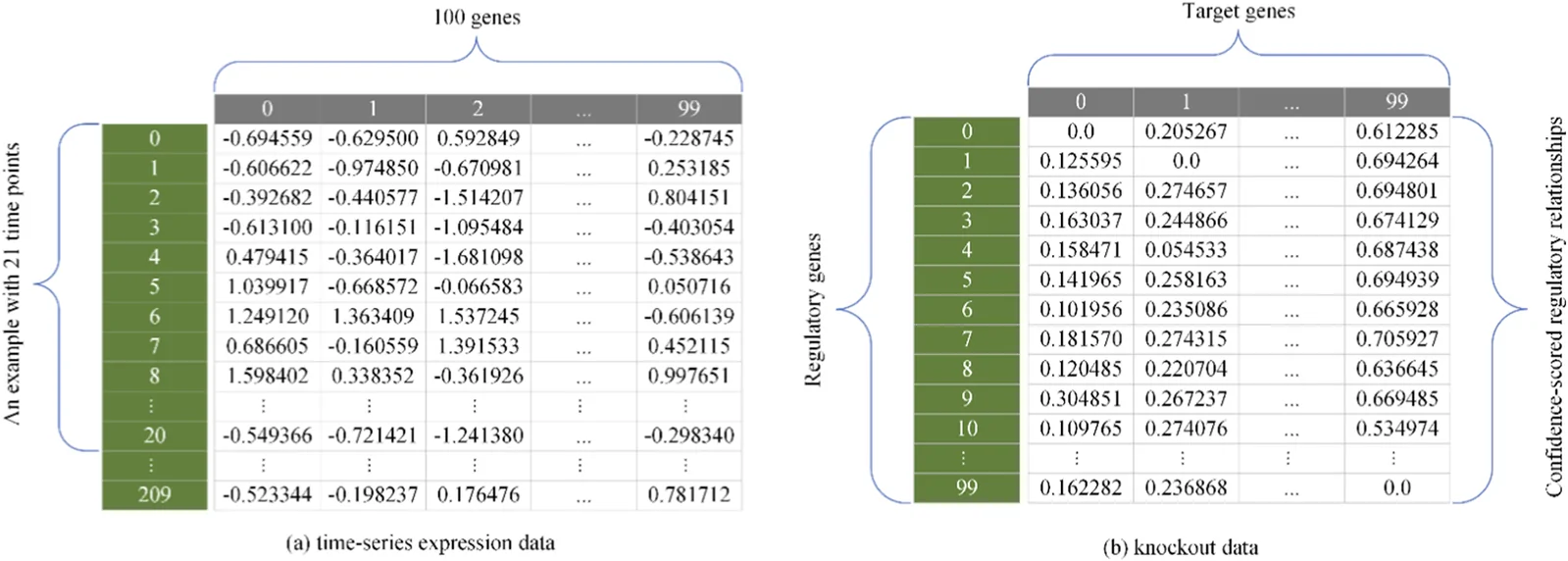
The expanded table contents represent two categories of data derived from Network 1 of the DREAM4 *in silico* 100-gene challenge: **(a)** time-series expression profiles and **(b)** knockout measurements.

Here, we take Network 1 of the DREAM4 *in silico* 100-gene challenge as an illustrative example. As depicted in Figure 1, this network is accompanied by two data tables: Table (a) for time-series expression data and Table (b) for knockout data. In Table (a), each column (excluding the first row) contains 210 numeric entries, grouped into 10 consecutive samples of 21 time points each. This organization effectively captures the temporal dynamics of gene expression. Table (b) reports the confidence scores of candidate regulatory interactions, where the row and column indices identify the regulator and target genes, respectively. Each cell entry thus indicates the inferred strength or likelihood of a regulatory influence from the regulator to the target.

#### 2.1.2 *Escherichia coli* datasets

The *E. coli* (*Escherichia coli*) datasets provide comprehensive time-series expression profiles under a wide range of environmental perturbations. In this study, we selected the datasets corresponding to three distinct stress conditions—cold, heat, and oxidative stress—as our experimental benchmarks, as these conditions are known to elicit substantial transcriptional reprogramming. Following the preprocessing strategy described in (Zheng et al., 2019), which included normalization and quality control steps, we retained a total of 163 transcription factors (TFs) and 1,484 target genes for downstream analysis. The main statistical characteristics of the processed datasets are summarized in Table 2.

**TABLE 2:** The details of *Escherichia coli* datasets.

Network name	# target genes	# candidate regulators	# samples	# time points	# true edges
*E. coli* cold	1,484	163	3	8	3,080
*E. coli* heat	1,484	163	3	8	3,080
*E. coli* oxi	1,484	163	3	11	3,080

We obtained the gold standard datasets from two complementary sources: the DREAM5 challenge (Mordelet et al., 2012), which provides both *in silico* and experimentally supported benchmark networks, and RegulonDB (Gama-Castro et al., 2016), a curated database of experimentally verified regulatory interactions in *E. coli*. Together, these datasets serve as reliable references for evaluating the performance of our inferred networks.

### 2.2 Ensemble framework

A GRN can be represented as a directed graph $G\left(V,E\right)$ , where the set of nodes V corresponds to genes, and the set of directed edges E corresponds to regulatory relationships. Each directed edge $e_{ji}\in E$ represents regulation from gene j (the regulator) to gene i (the target gene). Inferring a GRN aims to reconstruct G from a gene expression matrix $M=\left[X_{1},\cdots ,X_{p}\right]$ with p genes and N samples, where the vector $X_{i}$ denotes the expression values of gene i across different samples. As mentioned above, a common approach is to solve the problem under an ensemble framework, where p subproblems can be formulated as:

$$X^{i}=f\left(X^{-i}\right)+\epsilon^{i},i\in \left(1,2,\cdots ,p\right)$$ (1)

Here, $X^{-i}$ represents the expression values of candidate regulators (e.g., all genes except gene i ) for target gene i , whose expression is denoted by $X^{i}$ ; f denotes a selected function (e.g., least-angle regression or random forest) that models the influence of candidate regulators on the target gene; and $\epsilon^{i}$ is the random noise term. Based on f , the confidence of each candidate regulator’s regulatory relationship to the target gene can be computed as the importance of the corresponding feature variable. Ultimately, all regulatory relationships inferred from the p subproblems are ranked by their confidence scores, and the top-ranked ones are used to construct the GRN.

For time-series data, the subproblem can be further formulated from Equation 1 as:

$$X_{t}^{i}=f_{t}\left(X_{t^{\prime }}^{-i}\right)+\epsilon_{t}^{i},i\in \left(1,2,\cdots ,p\right)$$ (2)

where $X_{t}^{i}$ is the expression value of gene i at time point t , $X_{t^{\prime }}^{-i}$ are the expression values of candidate regulators of gene i at one or more previous time points $t^{\prime }$ ( $t^{\prime }<t$ ), and $\epsilon_{t}^{i}$ denotes random noise at time t .

**FIGURE 2:**
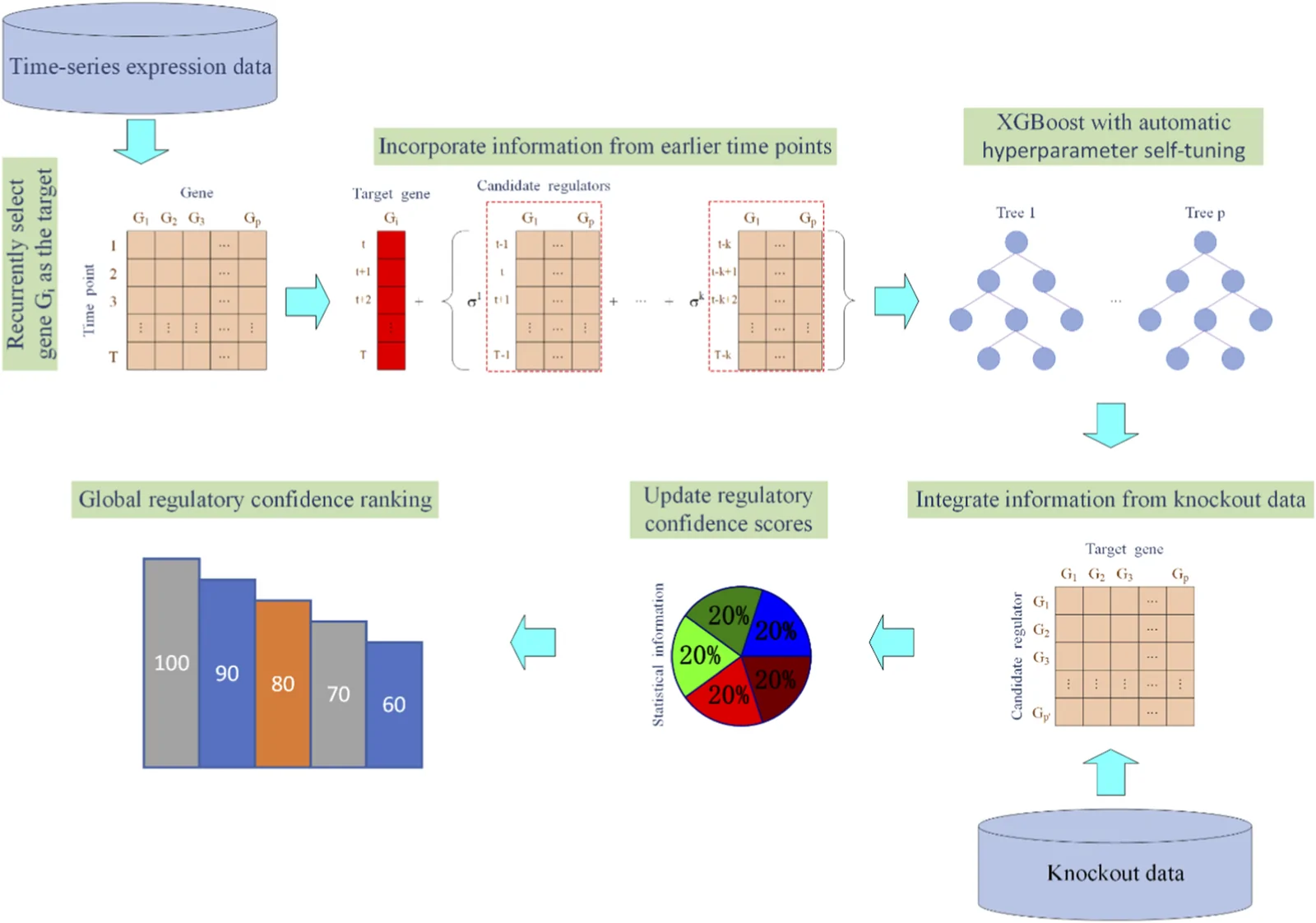
Schematic diagram of the proposed method. The procedure begins by iteratively selecting each gene as a target and formulating a feature selection subproblem that incorporates temporal information. This subproblem is then solved using a nonlinear boosting model equipped with automatic hyperparameter self-tuning. Subsequently, prior knockout data and regulatory statistics are integrated into the model, and the final gene regulatory network (GRN) is inferred from the global ranking of regulatory confidence scores.

The overall analysis workflow of this study is depicted in Figure 2. Specifically, we begin by iteratively selecting each gene as a target and constructing a feature selection subproblem that incorporates temporal information from earlier time points. Next, a nonlinear boosting model equipped with automatic hyperparameter self-tuning is applied to solve each subproblem, producing initial confidence scores for every candidate regulatory relationship. Subsequently, we incorporate prior knowledge derived from knockout experiments along with posterior information of regulatory statistics into the model, aiming to refine these preliminary scores. Ultimately, the GRN is reconstructed by ranking the refined confidence scores globally across all target genes and retaining the top-ranked interactions as the final predictions.

### 2.3 Incorporating information from earlier time points

In this study, we consider the accumulated impact of candidate regulators from k (here k≥1 ) previous time points on the target gene at time point t . Accordingly, we formulate the subproblem as:

$$X_{t}^{i}=f_{t}\left(U_{k}^{-i}\right)+\epsilon_{t}^{i},i\in \left(1,2,\cdots ,p\right)$$ (3)

where $U_{k}^{-i}$ denotes a vector collecting the accumulative expression values of the candidate regulators over the previous k time points, defined as:

$$U_{k}^{-i}=f_{a}\left(X_{t-1}^{-i},\cdots ,X_{t-k}^{-i},\delta \right)$$ (4)

Here, the value of k is determined by experimental optimization, $f_{a}$ represents the accumulation function, and 0<δ≤1 indicates a decay factor, reflecting the assumption that the influence of a time point is larger when it is closer to t . In this study, $f_{a}$ is simply defined as:

$$f_{a}\left(R_{-1},\cdots ,R_{-k},\delta \right)=\sum_{j=-k}^{-1}R_{j}\times \delta^{-j}$$ (5)

It should be noted that for most existing algorithms (e.g., GENIE-lag (Huynh-Thu, , 2012)), the time point $t^{\prime }$ in Equation 2 is set to t−1 , based on the assumption that the expression value of the target gene at time t is influenced only by the expression values of candidate regulators at the immediately preceding time point. In such cases, the subproblem is defined as:

$$X_{t}^{i}=f_{t}\left(X_{t-1}^{-i}\right)+\epsilon_{t}^{i},i\in \left(1,2,\cdots ,p\right)$$ (6)

Note that if k=1 , Equation 3 reduces to Equation 6. Thus, Equation 6 can be regarded as a special case of our method.

The recently proposed BiXGBoost (Zheng et al., 2019) also considers the impact of candidate regulators from k time points; however, it requires solving the subproblem k times to select the time point with the strongest influence. Moreover, the overall impact of candidate regulations is more likely to be the accumulation of effects over previous time points rather than the maximum single effect.

### 2.4 XGBoost model and its automatic hyperparameter self-tuning

#### 2.4.1 XGBoost model

XGBoost (eXtreme Gradient Boosting) is a scalable tree boosting system that has achieved state-of-the-art performance across a wide range of machine learning tasks (Chen and Guestrin, 2016). It builds an ensemble of regression trees in a stage-wise, additive manner by optimizing a regularized objective function that carefully balances predictive accuracy with model complexity. Key innovations include a sparsity-aware split-finding algorithm that efficiently handles missing values and one-hot-encoded categorical features; a theoretically justified weighted quantile sketch for approximate tree learning; and cache-aware prefetching to reduce memory access overhead during training. The system supports multiple split-finding strategies—including exact greedy, approximate global, and local methods—and enables out-of-core computation via block compression and disk sharding. XGBoost scales efficiently to billions of examples on a single machine and can be further distributed across clusters for even larger datasets. Its combination of efficiency, flexibility, and robustness has made it a widely adopted tool in both data science competitions and large-scale production pipelines.

**FIGURE 3:**
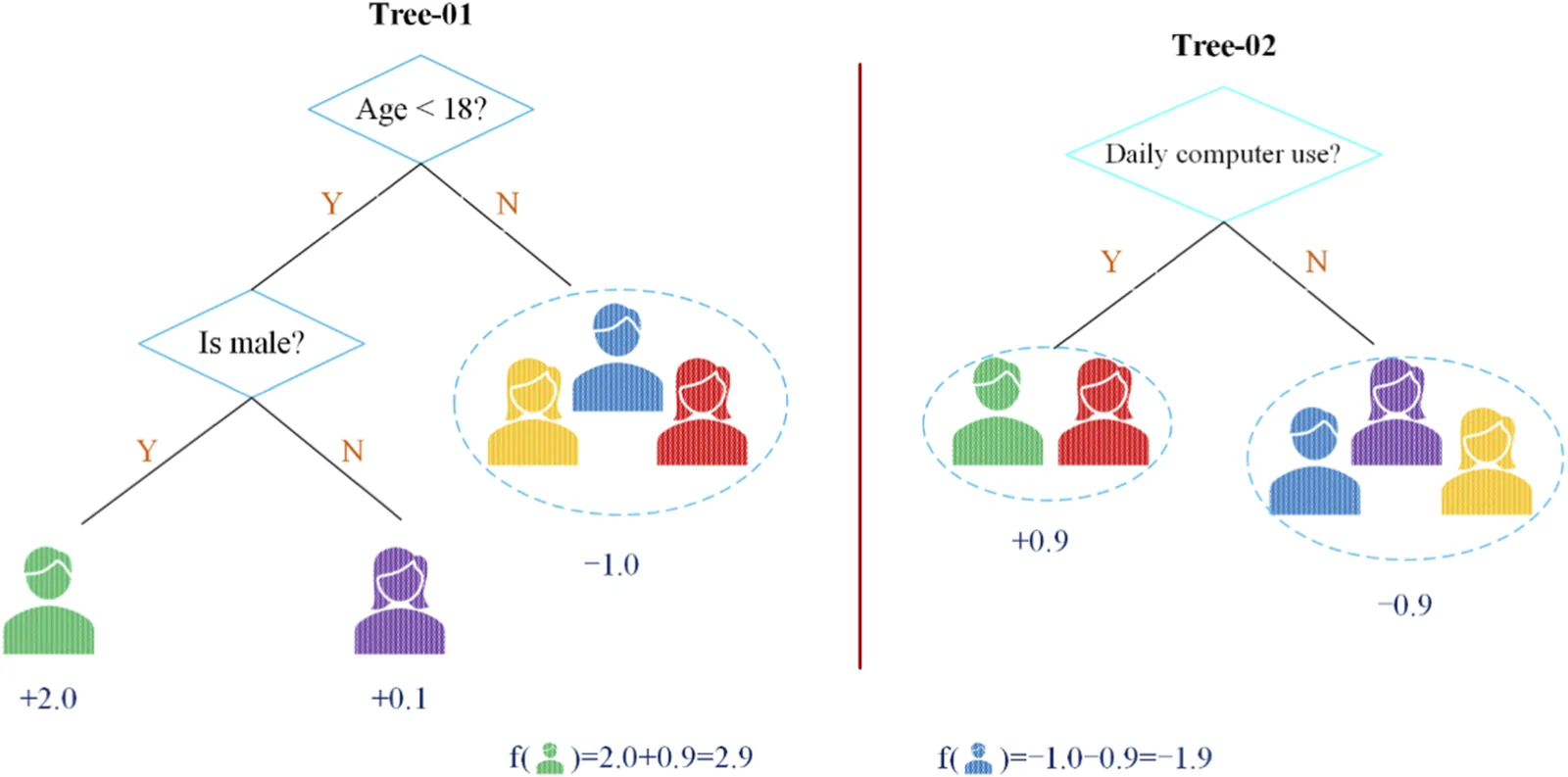
Illustration of a tree ensemble model. For a given input example, each tree contributes a score, and the final prediction is obtained by summing these scores across all trees in the ensemble.

Figure 3 illustrates a tree ensemble model in XGBoost, which combines multiple regression trees to make predictions. In this example, the ensemble consists of two regression trees. For a given input instance—such as the green male adolescent shown in the figure—each tree applies a series of learned decision rules to route the instance to one of its leaf nodes. The first tree assigns a score of +2.0, while the second contributes +0.9. The final prediction is obtained by summing these individual tree outputs, yielding a total score of 2.9 for this instance. This additive ensemble approach not only enables the model to capture complex, nonlinear patterns in the data but also preserves interpretability at the tree level.

#### 2.4.2 Automatic self-tuning of hyperparameters

XGBoost is characterized by a large set of hyperparameters, which provide considerable modeling flexibility but also introduce the practical challenge of parameter tuning. The performance of XGBoost is highly sensitive to these hyperparameters, making careful selection essential for achieving optimal results. The principal hyperparameters and their corresponding descriptions are summarized in Table 3 for easy reference.

**TABLE 3:** Key hyperparameters of XGBoost.

Hyperparameter	Description
max_depth	Maximum depth of a tree. Deeper trees can model more complex patterns but increase the risk of overfitting
learning_rate	Step size shrinkage. Reduces each tree’s contribution, preventing overfitting and enabling gradual learning
subsample	Fraction of training samples to be randomly sampled for each tree. Lower values help prevent overfitting
colsample_bytree	Fraction of features to be randomly sampled for each tree. Adds randomness and reduces overfitting
alpha	L1 regularization term on weights. Encourages sparsity in feature weights, leading to simpler models
lambda	L2 regularization term on weights. Smooths the leaf weights and helps prevent overfitting
min_child_weight	Minimum sum of instance weights (Hessian) required in a child leaf. Larger values increase conservatism
gamma	Minimum loss reduction required to make a further partition on a leaf node

Manual tuning of XGBoost hyperparameters is a labor-intensive process that critically influences model performance. Improper settings can severely degrade predictive accuracy, especially in challenging tasks such as GRN reconstruction, where the underlying regulatory logic is often nonlinear and highly context-dependent.

Automatic hyperparameter tuning provides an effective remedy. It not only frees researchers from tedious manual exploration but also enables a more principled search for optimal configurations. More importantly, it facilitates gene-specific hyperparameter optimization—each target gene receives a tailored set of hyperparameters that best fits its own expression dynamics and regulatory dependencies. This individualized strategy consistently outperforms the conventional approach of applying a single parameter configuration across all genes, leading to significant gains in inference accuracy.

Thus, automatic tuning offers dual benefits: it reduces human workload while improving the model’s capability to uncover genuine regulatory interactions. Overall, integrating automatic hyperparameter optimization into XGBoost significantly enhances its utility and reliability for genome-scale GRN reconstruction from expression data.

### 2.5 Integrating information from knockout data

#### 2.5.1 L2-norm-based normalization

Since each subproblem is solved independently using a boosting method, the resulting confidence scores of regulatory relationships are not directly comparable across different subproblems. To address this issue and enable consistent integration with knockout data, we apply an L2-norm-based normalization to standardize the weights across all candidate regulators for each target gene.

Specifically, for each subproblem corresponding to target gene i , the normalized weight ${\tilde{w}}_{ji}$ for candidate regulator j is computed as:

$${\tilde{w}}_{ji}=\frac{w_{ji}}{\sqrt{\sum_{j=1}^{p}w_{ji}^{2}}},j\neq i$$ (7)

where p denotes the number of candidate regulators for target gene i , and $w_{ji}$ represents the raw confidence score of the regulatory relationship from regulator j to target gene i .

This normalization ensures that the magnitude of weights becomes comparable across subproblems, thereby enabling fair and effective information fusion from all predicted regulatory interactions in the subsequent steps.

#### 2.5.2 Knockout-data-based confidence refinement

Some representative algorithms, such as HiDi (Deng et al., 2017) and iRafNet (Petralia et al., 2015), typically incorporate prior knowledge from additional data sources (e.g., gene expression profiles from knockout experiments) during the preprocessing stage. In contrast, we present a method that fuses prior information directly into our model—other data types can be integrated in a similar manner.

Without loss of generality, let $x_{ji}^{ko}$ denote the expression level of gene i after knocking out gene j . The corresponding prior information $I_{ji}^{ko}$ is then defined as:

$$I_{ji}^{ko}=\left\{\begin{array}{c} \frac{\left|x_{ji}^{ko}-\mu_{\cdot i}^{ko}\right|}{\sigma_{\cdot i}^{ko}},j\neq i\;and\;p_{r}=1\\ 1,p_{r}=0\; \end{array}\right.$$ (8)

where $p_{r}$ is a control parameter indicating whether to fuse the prior data information ( $p_{r}=1$ for fusion, $p_{r}=0$ otherwise). $\mu_{\cdot i}^{ko}$ is the mean expression level of gene i across all knockout experiments (where each regulator is iteratively knocked out only in the corresponding experiment), and $\sigma_{\cdot i}^{ko}$ is the corresponding standard deviation. The magnitude of $I_{ji}^{ko}$ reflects the significance of the deviation in $x_{ji}^{ko}$ relative to all knockout experiments: a larger value indicates greater significance. We assume that a large $I_{ji}^{ko}$ implies a high confidence in the regulatory relationship from gene j to gene i . In this study, the knockout data—the publicly available dataset shown in Figure 1b, where the matrix element corresponds to the $I_{ji}^{ko}$ value—is leveraged directly as the prior information. Based on this assumption, we update the global confidence scores ${\tilde{w}}_{ji}$ of all regulatory relationships as follows:

$${\hat{w}}_{ji}={\tilde{w}}_{ji}*I_{ji}^{ko},j\neq i$$ (9)

where ${\tilde{w}}_{ji}$ denotes the confidence score of the regulation from gene j to gene i , obtained through feature importance evaluation from the boosting method and then normalized using L2 norm. It should be noted that other data types (e.g., knockdown data) can be integrated in a similar way.

### 2.6 Updating regulatory confidence scores using statistical information

Furthermore, we employ a statistical technique to refine the weights ${\hat{w}}_{ji}$ . This approach is motivated by the hypothesis that if a candidate regulator j regulates multiple target genes, it is likely an important regulator; accordingly, the confidence scores of all regulatory relationships involving this regulator should be increased. Based on this rationale, we update the weights for each candidate regulator j as follows:

$${\bar{w}}_{ji}=\sigma_{j}^{2}\cdot {\hat{w}}_{ji,}\;i=1,2,\cdots ,p,and\;j\neq i$$ (10)

where $\sigma_{j}^{2}$ denotes the variance of all weights ${\hat{w}}_{ji}$ associated with candidate regulator j . Note that gene regulatory networks are typically sparse, meaning that most weights ${\hat{w}}_{ji}$ for a given regulator j are small. Consequently, a relatively large value of $\sigma_{j}^{2}$ indicates that several confidence scores for regulator j are substantially larger than the rest, suggesting that this regulator is likely to control the corresponding target genes.

Ultimately, we derive a gene regulatory matrix where each row corresponds to a candidate regulator, each column to a target gene, and each entry to the confidence score of the corresponding regulatory interaction. All entries are then flattened into a single list and sorted in descending order, with higher values indicating stronger potential regulatory links. Based on this ranking, we select the top entries as predicted regulatory edges to reconstruct the final GRN. This ranking-based procedure effectively prioritizes the most credible interactions while filtering out spurious ones.

## 3 Experimental results and data analysis

### 3.1 Evaluation criteria and comparison methods

To comprehensively evaluate the performance of the proposed method, we employed two widely used evaluation metrics—AUROC and AUPR—and benchmarked it against eight state-of-the-art methods spanning a diverse range of inference paradigms.

#### 3.1.1 Evaluation metrics

To evaluate the performance of our algorithm, we adopt two widely used metrics: the area under the receiver operating characteristic (AUROC) curve and the area under the precision-recall (AUPR) curve.

First, we compute the fundamental classification quantities: true positives (TP), true negatives (TN), false positives (FP), and false negatives (FN). Based on these, we derive the true positive rate (TPR) and the false positive rate (FPR) as follows:

$$TPR=\frac{TP}{TP+FN}$$ (11)

$$FPR=\frac{FP}{FP+TN}$$ (12)

TPR and FPR serve as the vertical and horizontal coordinates of the ROC curve, respectively. The AUROC is then calculated as the area under this curve, providing a measure of the model’s overall discriminative ability across all classification thresholds.

For AUPR, we first compute precision and recall:

$$Precision=\frac{TP}{TP+FP}$$ (13)

$$Recall=\frac{TP}{TP+FN}$$ (14)

AUPR is defined as the area under the precision-recall curve. This metric is particularly informative when dealing with imbalanced datasets, as it focuses on the performance of the positive class. Both metrics are computed from the same set of TP, TN, FP, and FN values derived from the model’s predictions.

#### 3.1.2 Baseline methods

To ensure a comprehensive and unbiased evaluation, we selected eight state-of-the-art methods as baselines, covering a broad spectrum of GRN inference paradigms—ranging from network topology-based approaches (e.g., HiDi (Deng et al., 2017), PFBNet (Che et al., 2020), NSRGRN (Liu et al., 2023)) to feature selection-based methods (e.g., GENIE-lag (Huynh-Thu, , 2012), Jump3 (Huynh-Thu and Sanguinetti, 2015), BiXGBoost (Zheng et al., 2019), iRafNet (Petralia et al., 2015)), as well as the overall winner of the DREAM challenge (Pinna et al., 2010). This diverse collection enables a fair and rigorous comparison across different methodological families, and allows us to assess the relative strengths and weaknesses of our proposed method in a variety of contexts.

### 3.2 Boundary setting for self-tuning hyperparameters

Compared with manual tuning, XGBoost with hyperparameter self-tuning offers several key advantages: it eliminates laborious trial-and-error, automatically tailors hyperparameters to each target gene, and significantly enhances model performance—especially in complex tasks such as gene regulatory network inference, where optimal configurations vary across regulators, thereby yielding more robust predictions.

Determining the hyperparameter range in XGBoost self-tuning is a critical task that warrants careful consideration. A range that is too broad tends to degrade the performance of gene regulatory inference markedly, often introducing instability. Conversely, a range that is overly narrow yields performance comparable to that of fixed hyperparameters, thereby diminishing the benefits of self-tuning. A range that is biased—i.e., not centered near the optimal region—can cause a substantial drop in inference accuracy, sometimes even underperforming fixed hyperparameters. An ideal range should first encompass the values that lead to peak performance, while also accounting for practical constraints such as computational cost. Only by carefully balancing these factors can a suitable hyperparameter range be identified.

**FIGURE 4:**
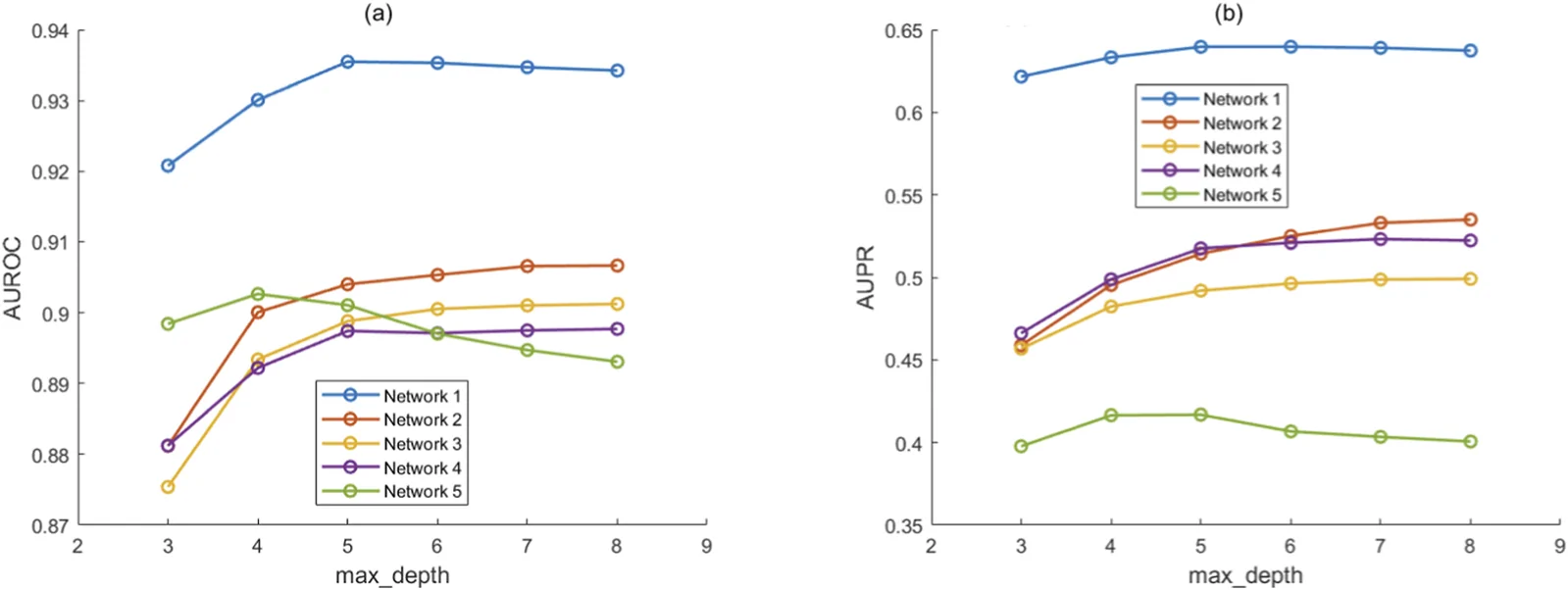
AUROC and AUPR performance on the DREAM4 networks as a function of max⁡_depth under the boundary determination strategy. Numerical performance data are available in the supplementary tables. **(a)** AUROC vs. max_depth on DREAM4 Networks. **(b)** AUPR vs. max_depth on DREAM4 Networks.

We take the determination of the self-tuning range for the hyperparameter max⁡_depth as an illustrative example. First, we fix the initial ranges of all other hyperparameters (see Table 3) – these ranges need not be precisely optimal, only reasonably non-extreme. Then, we let max⁡_depth take discrete values sequentially from the set $\left\{3,4,5,6,7,8\right\}$ . Cases with max⁡_depth≤2 or max⁡_depth≥9 are excluded from our evaluation, as the former results in unsatisfactory performance and the latter incurs prohibitive runtime overhead. For each candidate value, we compute and record the corresponding gene regulatory inference performance (AUROC and AUPR), as shown in Figure 4.

As shown in the figure, the inference performance (measured by both AUROC and AUPR) nearly reaches its peak when max⁡_depth is set to 5, 6, or 7. Although increasing max⁡_depth beyond 7 leads to only marginal performance gains, it substantially increases the execution time, making those settings less practical. Therefore, we set the self-tuning range as *'max_depth’: randint(5, 7)*, allowing the tuning mechanism to choose among these three values and strike a balanced trade-off between predictive accuracy and computational efficiency.

The self-tuning ranges for the other hyperparameters in Table 3 can be determined in a similar manner. Owing to space constraints and to avoid unnecessary repetition, we do not elaborate further here; instead, the exact ranges for each hyperparameter are given in the supplementary Python code. This consistent approach is applied to all hyperparameters, thereby maintaining a cohesive tuning framework.

### 3.3 Performance evaluation on the DREAM4 datasets

#### 3.3.1 Without knockout data

**FIGURE 5:**
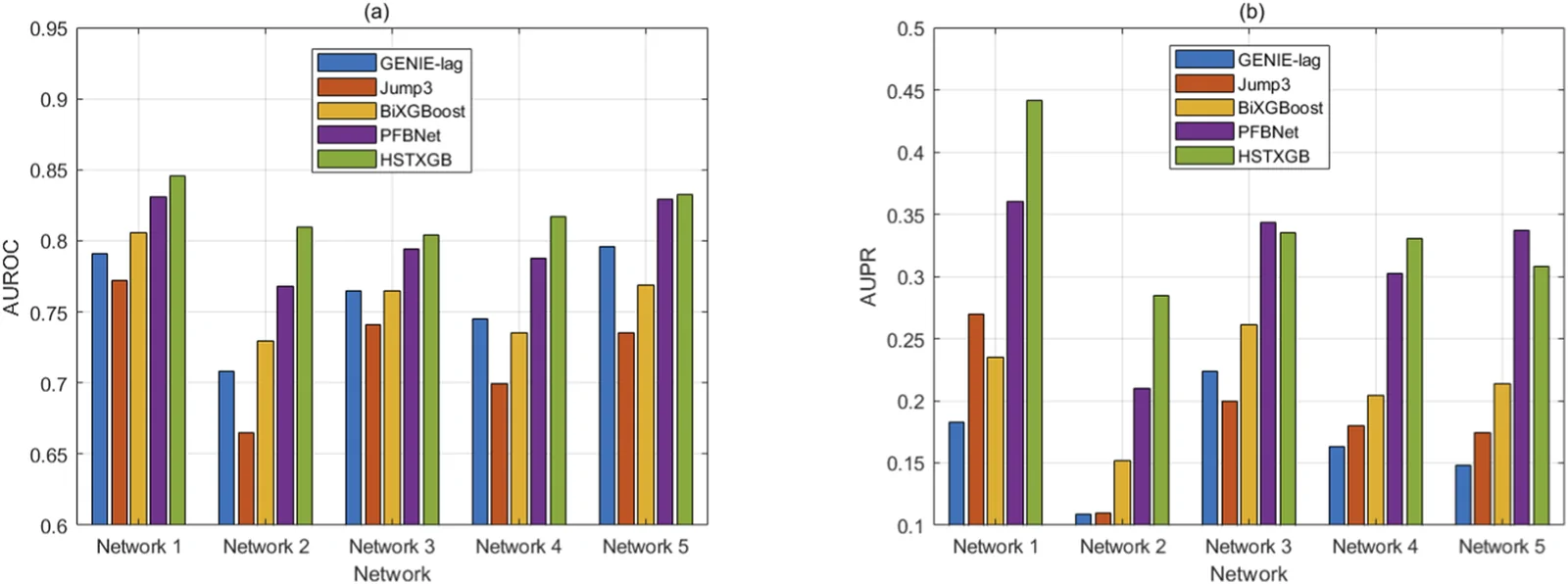
AUROC and AUPR performance on the DREAM4 networks without utilizing knockout data. Numerical performance data are provided in the supplementary tables. **(a)** AUROC on DREAM4 datasets without knockout data. **(b)** AUPR on DREAM4 datasets without knockout data.

Since GENIE-lag, Jump3, and BiXGBoost do not support the integration of knockout data, we configured HSTXGB without fusing any prior information, ensuring a fair basis for comparison. Figure 5 shows the comparative results of these methods on the DREAM4 *in silico* 100-gene challenge datasets.

As shown in Figure 5a, HSTXGB consistently achieves the highest AUROC across all five networks of the DREAM4 benchmark. Specifically, compared with the next best performing method, HSTXGB improves AUROC by 1.8% (Network 1), 5.5% (Network 2), 1.3% (Network 3), 3.7% (Network 4), and 0.5% (Network 5). These consistent gains clearly demonstrate the robustness and effectiveness of our approach under the AUROC metric.

For the AUPR metric (Figure 5b), HSTXGB surpasses the second-best performer on three networks, achieving improvements of 22.6% (Network 1), 35.6% (Network 2), and 9.4% (Network 4). On Networks 3 and 5, HSTXGB shows slightly lower AUPR than PFBNet, with differences of 2.2% and 8.7%, respectively. Nevertheless, apart from these two cases, HSTXGB remains highly competitive overall and consistently performs well on the majority of networks.

#### 3.3.2 With knockout data

**FIGURE 6:**
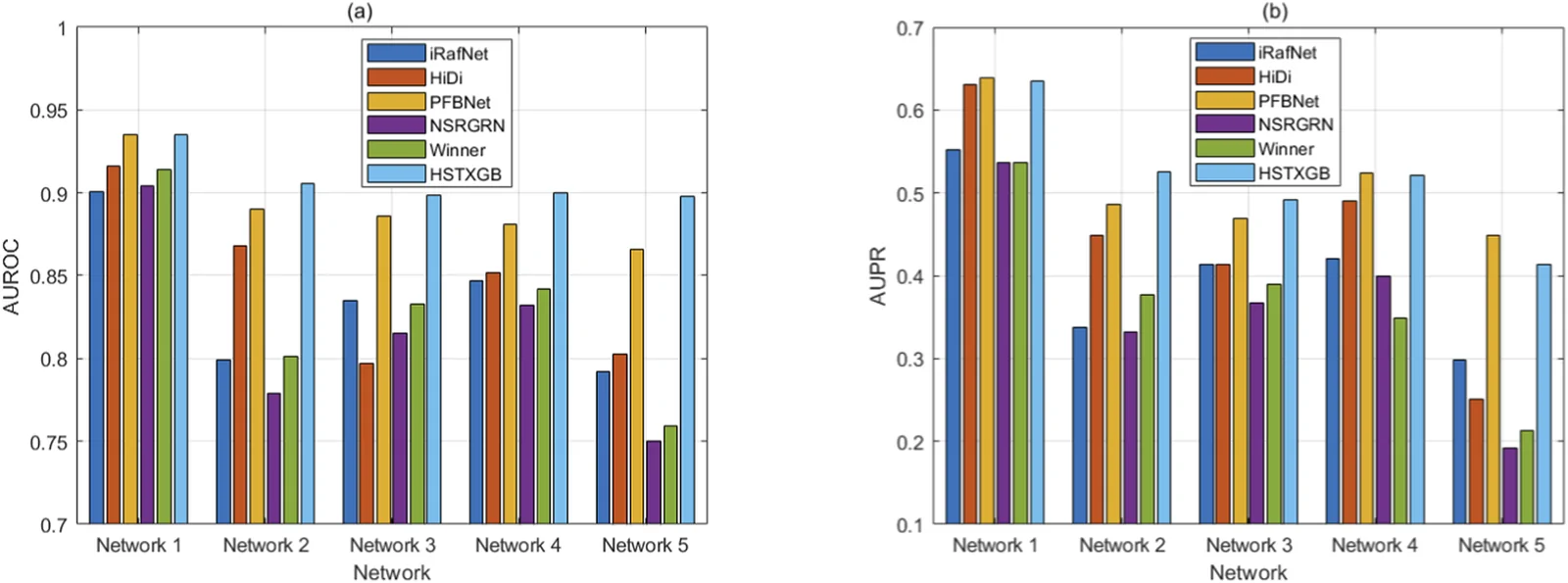
Comparative AUROC and AUPR performance on the DREAM4 networks with the incorporation of knockout data. Detailed numerical performance data are available in the supplementary tables. **(a)** AUROC on DREAM4 datasets with knockout data. **(b)** AUPR on DREAM4 datasets with knockout data.

To comprehensively evaluate HSTXGB, we compared it with five state-of-the-art methods—iRafNet, HiDi, PFBNet, NSRGRN, and the winner of the DREAM4 *in silico* 100-gene challenge—each of which employs distinct strategies for integrating prior information from auxiliary data sources (e.g., knockout data). The comparative results are presented in Figure 6.

As shown in Figure 6a, HSTXGB consistently achieves highly competitive AUROC values across all five networks. Specifically, it matches PFBNet on Network 1 with the same top score of 0.935, and outperforms the next best method on Networks 2–5 by 1.8%, 1.4%, 2.2%, and 3.7%, respectively. Notably, on Network 5, HSTXGB attains 0.898, surpassing all other methods by a substantial margin.

In terms of the AUPR metric shown in Figure 6b, HSTXGB consistently demonstrates strong and robust overall performance. It exactly ties with PFBNet as the top performer on Networks 1 (0.64) and 4 (0.52). On Networks 2 and 3, HSTXGB achieves notable improvements of 8.2% and 5.0% over the second-best method, respectively. On Network 5, although HSTXGB (0.413) is slightly lower than PFBNet (0.448) by 7.8%, it still ranks among the top two methods.

A comparison between Figures 5, 6 clearly reveals that the substantial performance gains achieved through the incorporation of prior information underscore the critical role of data fusion in achieving accurate GRN inference. Moreover, the consistent improvements observed across both evaluation metrics and for the majority of networks further validate the effectiveness of our strategy to integrate knockout data into the HSTXGB framework.

### 3.4 Performance evaluation on the *Escherichia coli* datasets

To further assess the robustness of our HSTXGB algorithm, we conducted additional experiments on the *E. coli* datasets, benchmarking it against four other methods: GENIE-lag, Jump3, BiXGBoost, and PFBNet. It is worth noting that iRafNet, HiDi, and NSRGRN were excluded, as the *E. coli* datasets only provide time-series expression data without knockout information. Following the same experimental setup, we adopted AUROC and AUPR as the evaluation metrics and ran all algorithms with their default parameter settings.

**FIGURE 7:**
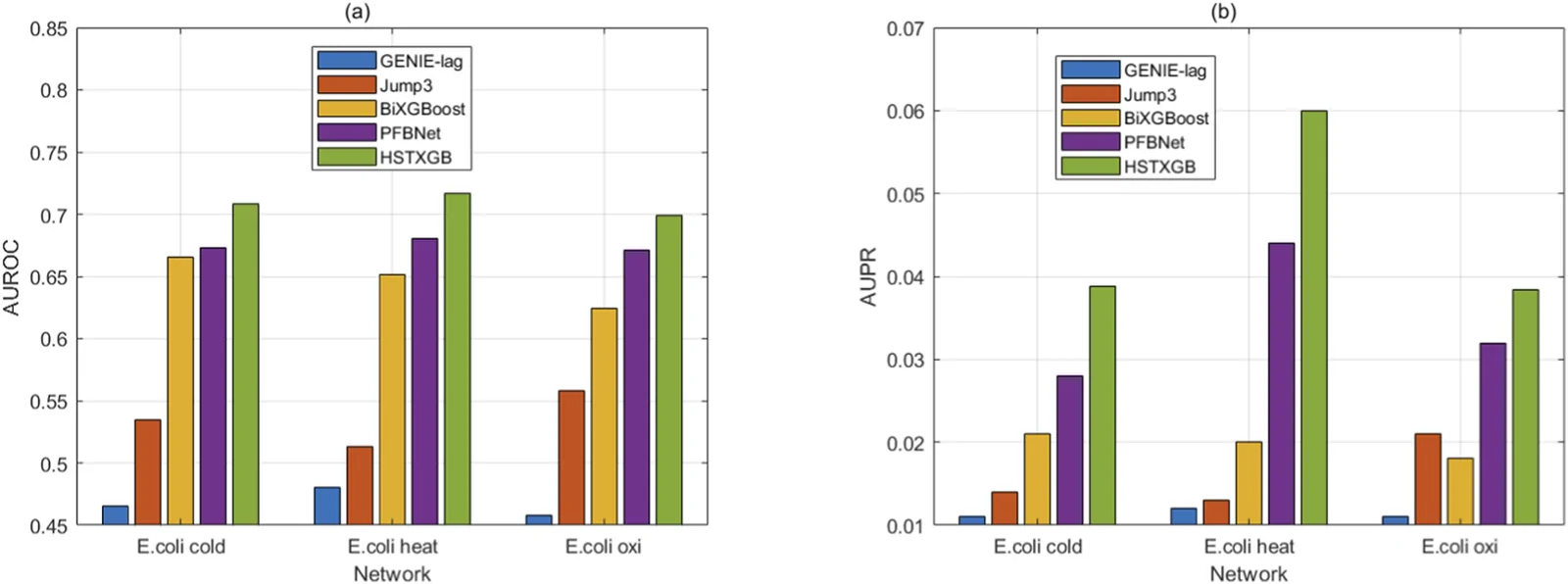
Comparative AUROC and AUPR performance on the *Escherichia coli* networks without knockout data. Detailed numerical performance data are available in the supplementary tables. **(a)** AUROC on E.coli datasets (without knockout data). **(b)** AUPR on E.coli datasets (without knockout data).

The comparative results are presented in Figure 7. As shown in Figure 7a, HSTXGB consistently achieves the highest AUROC across all three environmental stress conditions. Specifically, compared with the second-best method, HSTXGB yields AUROC improvements of 5.3% (Cold), 5.4% (Heat), and 4.2% (Oxidative-stress). These consistent gains clearly demonstrate the robustness of HSTXGB under diverse biological perturbations.

In terms of AUPR (Figure 7b), HSTXGB once again outperforms all competing methods. Specifically, its average AUPR is 38.6%, 36.2%, and 19.9% higher than that of the second-best algorithm across the Cold, Heat, and Oxidative-stress conditions, respectively. Such substantial and consistent improvements clearly underscore the effectiveness of HSTXGB in accurately identifying regulatory relationships, even when only time-series expression data are available.

Overall, these experimental results collectively and strongly indicate that HSTXGB is not only consistently effective on synthetic benchmark networks (e.g., the DREAM4 datasets) but also highly suitable for reconstructing complex, large-scale gene regulatory networks from real-world time-series expression data. Its robust performance across diverse experimental conditions and data types further underscores its practical utility and generalizability, positioning it as a reliable and versatile tool for uncovering regulatory interactions in various biological contexts.

## 4 Conclusions and future work

We propose HSTXGB, a feature selection-based method for GRN inference from gene expression data. Unlike previous approaches, HSTXGB leverages the nonlinear modeling capability of XGBoost while integrating prior knowledge (e.g., knockout data) and posterior statistics (e.g., regulation probabilities) via a hyperparameter self-tuning strategy. First, we iteratively select each gene as a target and formulate a feature selection subproblem that incorporates temporal information from previous time points. Next, we apply a nonlinear boosting model with automatic hyperparameter self-tuning to obtain initial confidence scores for candidate regulatory relationships. These scores are then refined by fusing knockout-derived prior information and regulatory statistics. Finally, the GRN is constructed based on the global ranking of the refined confidences across all target genes. Experimental results on DREAM challenge and *E. coli* benchmark datasets demonstrate that HSTXGB significantly outperforms eight state-of-the-art methods, confirming its effectiveness on both synthetic networks and real time-series data for large-scale GRN reconstruction.

We acknowledge several limitations in the current study and offer two perspectives for future improvement. First, although XGBoost is effective for GRN inference, its greedy tree-building strategy may miss subtle regulatory interactions, and it struggles with ultra-high-dimensional data where regulators far outnumber samples. Moreover, XGBoost requires extensive hyperparameter tuning to avoid overfitting. Future work could explore Unbiased Gradient Boosting Decision Tree (UnbiasedGBM) with more advanced regularization and feature selection. We anticipate that UnbiasedGBM variants incorporating domain-specific biological priors could better capture the sparse and hierarchical nature of gene regulation, improving both interpretability and accuracy. Second, we intend to propose a two-step strategy for GRN reconstruction. In the first step, a boosting model ranks candidate regulators for each target gene; regulators with near-zero confidence scores are filtered out. In the second step, the model is reapplied to the reduced candidate set to generate refined scores. The top-ranked regulators are then retained as final predictions. This sequential pruning and re-ranking reduces false positives and computational burden, leading to more accurate and efficient GRN inference.

## Data Availability

The complete open-source Python source code, experimental datasets, and Supplementary Material accompanying this study are publicly accessible via the GitHub repository at: https://github.com/MingqingHuang-SHU/HSTXGB. Users are encouraged to freely download the repository to reproduce the results, verify the analyses, or adapt the code for their own related research.
